# Optimization of Ultrasonic Extraction Parameters for Polyphenolic-Rich Extract from Peanut Shells and Its Application in Functional Yogurt

**DOI:** 10.3390/molecules31071066

**Published:** 2026-03-24

**Authors:** Tamara Tultabayeva, Umyt Zhumanova, Bakhtiyar Tultabayev, Aruzhan Shoman, Assem Sagandyk, Aknur Muldasheva, Daulet Aiken, Nuray Battalova, Mukhtar Tultabayev, Nurtore Akzhanov

**Affiliations:** 1 University of California, 1 Shields Ave, Davis, CA 95616, USA; 2 S. Seifullin Kazakh Agrotechnical Research University, Astana 010011, Kazakhstan; 3 Agro Tech Scientific and Innovation Center, Astana IT University, Astana 010000, Kazakhstan; 4 Department of Technology and Standardization, K. Kulazhanov Kazakh University of Technology and Business, Astana 010000, Kazakhstan; 5 Eurasian National University Named After L.N. Gumilyov, Astana 010000, Kazakhstan

**Keywords:** ultrasonic extraction, polyphenols, peanut shells, functional yogurt

## Abstract

The aim of this study was to optimize the parameters of ultrasonic extraction of polyphenolic compounds from peanut shells and to evaluate the feasibility of using the obtained extract in the development of functional yogurt. The extraction factors considered were the ethanol concentration, particle size of peanut shells, and extraction time. Process optimization was performed using response surface methodology based on a second-order central composite design. Extraction yield and total polyphenol content were selected as the optimization criteria. The optimal ultrasonic extraction conditions were determined as an ethanol concentration of approximately 70% ethanol, 300 μm particle size, and 53 min. Under these conditions, the predicted extraction yield was 9.05% and the total polyphenol content reached 95.15 mg GAE/g of dry extract. The extract obtained under the optimal conditions was used to fortify yogurt at concentrations of 0.25%, 0.5%, and 0.75%. Physicochemical analysis showed that the addition of peanut shell polyphenol extract increased the water-holding capacity and reduced syneresis of yogurt during storage compared with the control sample. Changes in pH and titratable acidity remained within the typical ranges for fermented dairy products. The results confirm the potential of peanut shells as a promising source of polyphenolic compounds and demonstrate the feasibility of using the optimized extract in the development of functional fermented dairy products.

## 1. Introduction

Modern food science increasingly considers polyphenols as key plant-derived bioactive compounds capable of enhancing the antioxidant and functional properties of foods, as well as creating added value through the utilization of plant raw materials and agro-industrial by-products. 

The growing interest in plant-based functional foods is directly associated with the presence of polyphenolic compounds in food matrices. Sridhar A. et al. (2021) [1] highlighted that polyphenol extraction is challenging, as methods must preserve extract and raw material quality, with intensified techniques like ultrasound-assisted extraction (UAE) offering higher efficiency, lower energy use, and better extract quality compared to conventional approaches (maceration, Soxhlet, etc.). Key parameters include solvent type, temperature, and particle size.

Recent studies confirm luteolin enrichment in peanut shell fractions with enhanced antioxidant and AChE inhibitory activities (Tran T. N. et al., 2025) [2], supporting potential functional benefits.

The impact of temperature and extraction conditions on the stability of polyphenolic compounds has been thoroughly examined by Antony A. et al., who analyzed the following apparent contradiction: polyphenols are generally regarded as thermolabile, yet subcritical water extractions at temperatures up to 200 °C sometimes yield higher phenolic content without severe degradation. This may result from matrix-specific effects, selective extraction of stable fractions, Maillard reaction products formation, oxidation, or light exposure. The study also noted limitations of the Folin–Ciocalteu method, including poor correlation with actual antioxidant activity.

Particular attention in the literature is given to ultrasonic-assisted extraction (UAE) as one of the most promising methods for intensifying polyphenol extraction processes. Dzah C. S. et al. [3] note that despite its widespread use, comprehensive parameter reviews are rare. Temperatures above 50 °C risk degradation, lower frequencies better disrupt cell walls, and higher power increases yield only to a threshold—beyond which free radical formation (especially in aqueous media) may degrade polyphenols, highlighting the need for precise optimization to avoid contradictions between yield gain and compound stability.

Significant attention in the literature has been given to peanut processing by-products. Dean L. L. [4] notes that peanut skins and shells, traditionally considered waste, are rich sources of polyphenolic compounds and can be utilized in both food and non-food applications. Summarizing these findings, Toomer O. T. [5] emphasizes that the scale of global peanut production generates substantial volumes of by-products, which have high potential as antioxidant, functional, and antimicrobial ingredients. The chemical composition and varietal variability of phenolic compounds in peanut shells have been thoroughly investigated by Kumar S. P. J. et al. [6], who confirmed the presence of phenolic acids and flavonoids using FTIR and HPLC techniques, and highlighted the significant influence of cultivar and solvent on extraction efficiency.

While peanut skins have received more focus (e.g., Sorita G. D. et al. [7] on proanthocyanidins and green high-pressure methods), studies on shells are fewer. Imran A. et al. showed that ethanol-based UAE yields a higher polyphenol content and antioxidant activity than conventional methods, with notable luteolin recovery. Liao J. et al. optimized UAE for flavonoids in shells (including ethanol concentration and particle size) using kinetic models. Kang J. W. et al. [8] confirmed antioxidant and anti-inflammatory effects linked to luteolin in UAE shell extracts.

The phenolic fraction of peanut skins, rich in proanthocyanidins, is considered by Sorita G. D. et al. [7] as a promising functional ingredient for the food and pharmaceutical industries, with the authors emphasizing the importance of “green” high-pressure extraction methods and encapsulation to enhance the bioavailability and stability of the compounds. Studies directly focused on peanut shells have been conducted by Imran A. et al., who demonstrated that ultrasonic extraction using ethanol provides higher polyphenol yield and antioxidant activity compared to conventional methods, while also facilitating the extraction of significant amounts of luteolin. A more advanced approach to fractionating phenolic compounds from peanut shells was proposed by Tran T. N. et al. [2], who showed that sequential fractionation of extracts allows the obtaining of flavonoid-enriched fractions with high antioxidant activity and pronounced acetylcholinesterase inhibitory effects.

Despite promising results from UAE on peanut by-products, a clear research gap persists: few studies have applied multi-criteria response surface methodology (RSM) with desirability function optimization specifically to peanut shells (as opposed to skins), simultaneously maximizing both extraction yield and total polyphenol content while incorporating particle size as a key factor. Moreover, the technological application of such optimized shell extracts in fermented dairy products like yogurt—to improve structural stability (e.g., water-holding capacity and reduced syneresis) via protein–polyphenol interactions—remains largely unexplored.

We hypothesize that RSM-guided optimization of UAE parameters (moderate ethanol concentration ~70%, fine particle size 300–500 μm, and controlled time ~50 min) will enable efficient recovery of a polyphenol-rich extract from peanut shells, and that moderate fortification (0.25–0.75%) in yogurt will enhance physicochemical stability during storage without disrupting fermentation parameters (pH and titratable acidity within typical ranges).

Accordingly, the objectives of this study were to optimize UAE parameters using a central composite design and desirability function to maximize extraction yield and total polyphenol content; produce and characterize the extract under optimal conditions; and evaluate its effects on key physicochemical properties (water-holding capacity, syneresis, pH, titratable acidity) of fortified yogurt during refrigerated storage.

## 2. Results

### 2.1. Mathematical Modeling of the Ultrasonic Extraction Process

Based on experimental data obtained according to the second-order rotatable central composite design, a mathematical model was developed to describe the influence of technological parameters of ultrasonic extraction on the yield of extract from peanut shells. The independent factors were solvent concentration (C, %), particle size of ground shells (K, μm), and extraction time (t, min). The response variable of the model was extraction yield (Y, %).

A second-order polynomial equation was used to approximate the experimental data. The regression equation in coded factor values is as follows:

Y_1_ = b_0_ + b_1_X_1_ + b_2_X_2_ + b_3_X_3_ + b_12_X_1_X_2_ + b_13_X_1_X_3_ + b_23_X_2_X_3_ + b_11_X_1_^2^ + b_22_X_2_^2^ + b_33_X_3_^2^

Taking into account the calculated coefficients, the regression equation in coded factor values is expressed as follows:

Y_1_ = 8.82 + 0.522X_1_ − 0.357X_2_ − 0.069X_3_ − 0.175X_1_X_2_ + 0.200X_1_X_3_ − 0.175X_2_X_3_ − 0.284X_1_^2^ − 0.160X_2_^2^ − 0.196X_3_^2^

A positive coefficient for x_1_ indicates that increasing the solvent concentration within the studied range has a positive effect on the extraction yield. Negative coefficients for x_2_ and x_3_ suggest a decrease in extraction yield with increasing particle size and excessive extraction time, which may be associated with impaired mass transfer and possible degradation of the extracted components.

The negative quadratic coefficients **b_11_**, **b_22_**, and **b_33_** indicate the presence of well-defined extrema in the response function and confirm the appropriateness of using a second-order model to describe the process. Interactions between factors **x_1_x_2_**, **x_1_x_3_**, and **x_2_x_3_** exert a moderate influence on extraction yield, reflecting the complex nature of the extraction process.

For practical applications, the model was also expressed in natural factor values, allowing direct calculation of extraction yield for given technological parameters:

Y = −59.83 + 1.395C + 0.051K + 0.346t − 0.00035CK + 0.008Ct − 0.00035Kt − 0.01135C^2^ − 1.60 × 10^−5^K^2^ − 0.00783t^2^

Analysis of the coefficients in natural factor values confirms that solvent concentration has the greatest influence on extraction yield, while particle size and extraction time exhibit optimal values within the studied ranges.

The adequacy of the developed mathematical model was evaluated by comparing the reproducibility variance and the adequacy variance. The reproducibility variance was 0.1377, while the adequacy variance was 0.2016. This ratio indicates good agreement between experimental and predicted data, confirming the adequacy of the model.

**Figure 1:**
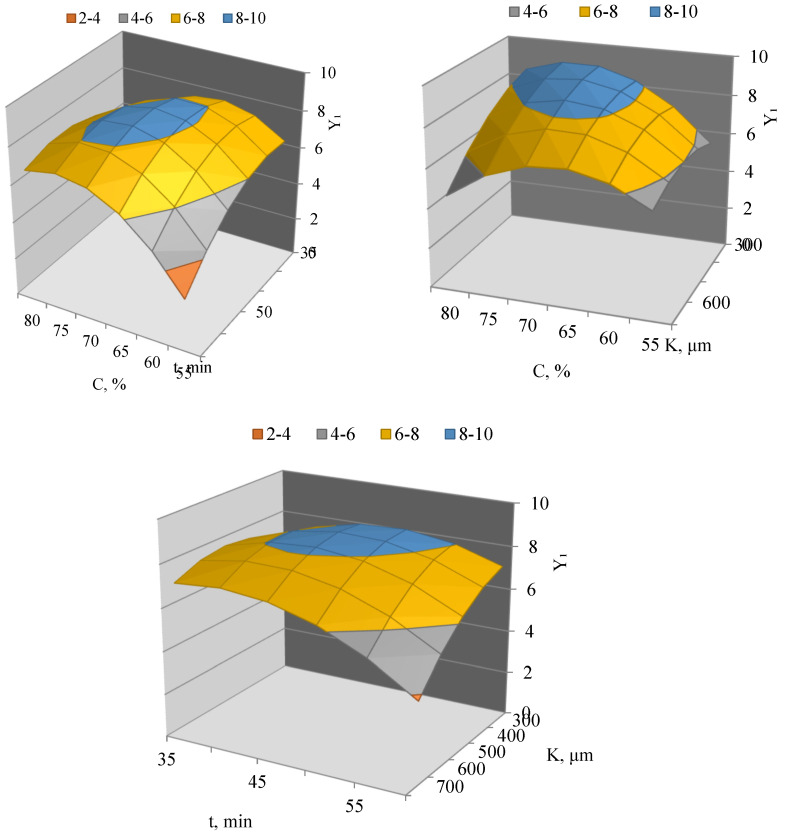
Three-dimensional response surfaces of extraction yield (Y_1_) depending on solvent concentration (C), particle size (K), and extraction time (t).

The confidence intervals for the regression coefficients were ±0.30 for the intercept, ±0.20 for linear and quadratic coefficients, and ±0.26 for interaction coefficients. Most of the significant coefficients exceed their corresponding confidence intervals, indicating their statistical significance and confirming the reliability of the observed trends (Figure 1).

Analysis of the three-dimensional response surfaces and the corresponding contour plots showed that the maximum extraction yield is achieved at intermediate particle sizes and extraction times combined with a higher solvent concentration. The response surface representing the combined effect of solvent concentration and particle size exhibits a pronounced maximum at an ethanol concentration of approximately 65–70% and a particle size of about 450–550 μm.

When analyzing the response surface in the “solvent concentration–extraction time” plane, it was observed that increasing the extraction time leads to an increase in yield only up to a certain limit, after which a decrease occurs. This may be associated with redistribution of the extracted compounds and reduced efficiency of ultrasonic treatment. A similar trend is observed when analyzing the effect of particle size and extraction time, with the optimum corresponding to intermediate values of the factors.

Based on the analysis of the mathematical model and response surfaces, the optimal parameters of the ultrasonic extraction process were determined to ensure maximum extraction yield. The optimal conditions are located in the central region of the experimental design and are characterized by a solvent concentration of approximately 65–70%, a particle size of about 500 μm, and an extraction time of around 45–50 min. These results confirm that ultrasonic extraction is an effective method for recovering bioactive components from peanut shells and allows for targeted control of extract yield through optimization of technological parameters.

### 2.2. Mathematical Modeling of the Extraction Process Based on Total Polyphenol Content

Using experimental data obtained from the second-order rotatable central composite design, a mathematical model was developed to describe the influence of technological parameters of ultrasonic extraction on the total polyphenol content in extracts from peanut shells. The independent factors were solvent concentration (C, %), particle size of ground peanut shells (K, μm), and extraction time (t, min). The response variable was total polyphenol content (Y_2_), expressed in mg of gallic acid equivalents per gram of dry extract (mg GAE/g).

A second-order polynomial model was used to approximate the experimental data. The generalized form of the regression equation in coded factor values is given as follows:

Y_2_ = b_0_ + b_1_x_1_ + b_2_x_2_ + b_3_x_3_ + b_12_x_1_x_2_ + b_13_x_1_x_3_ + b_23_x_2_x_3_ + b_11_x_1_^2^ + b_22_x_2_^2^ + b_33_x_3_^2^

Taking into account the calculated coefficients, the regression equation in coded factor values is expressed as follows:

Y_2_ = 78.715 + 5.740x_1_ − 0.658x_2_ + 1.005x_3_ − 0.175x_1_x_2_ − 0.025x_1_x_3_ − 0.650x_2_x_3_ + 2.150x_1_^2^ + 0.527x_2_^2^ + 0.421x_3_^2^

A positive value of the linear coefficient for **x_1_** indicates a significant effect of solvent concentration on the extraction of polyphenolic compounds. Increasing the ethanol concentration within the studied range promotes higher total polyphenol content in the extract. A positive coefficient for **x_3_** indicates the beneficial influence of ultrasonic treatment duration on polyphenol extraction efficiency, which is associated with intensified mass transfer and disruption of the plant cell structures.

A negative coefficient for **x_2_** indicates a decrease in polyphenol content with increasing particle size, which can be explained by the reduction in the specific surface area available for contact between the solvent and the raw material. The interaction between factors **x_2_x_3_** has a negative coefficient, indicating a decrease in polyphenol extraction efficiency when both particle size and extraction time are increased simultaneously.

The presence of positive quadratic coefficients **b_11_**, **b_22_**, and **b_33_** indicates pronounced curvature of the response surface and confirms the existence of extreme values of the response function within the studied range of factors.

For practical applications, the model was converted into natural factor values, allowing it to be used for predicting polyphenol content under real technological process conditions as follows:

Y_2_ = 365.394 − 9.811C + 0.022K − 0.600t − 0.00035CK − 0.001Ct − 0.00130Kt + 0.08599C^2^ + 5.27 × 10^−5^K^2^ + 0.01685t^2^

Analysis of the coefficients in natural factor values shows that solvent concentration is the dominant factor determining the level of polyphenol extraction. The effects of particle size and extraction time are nonlinear and exhibit optimal ranges within the studied values.

**Figure 2:**
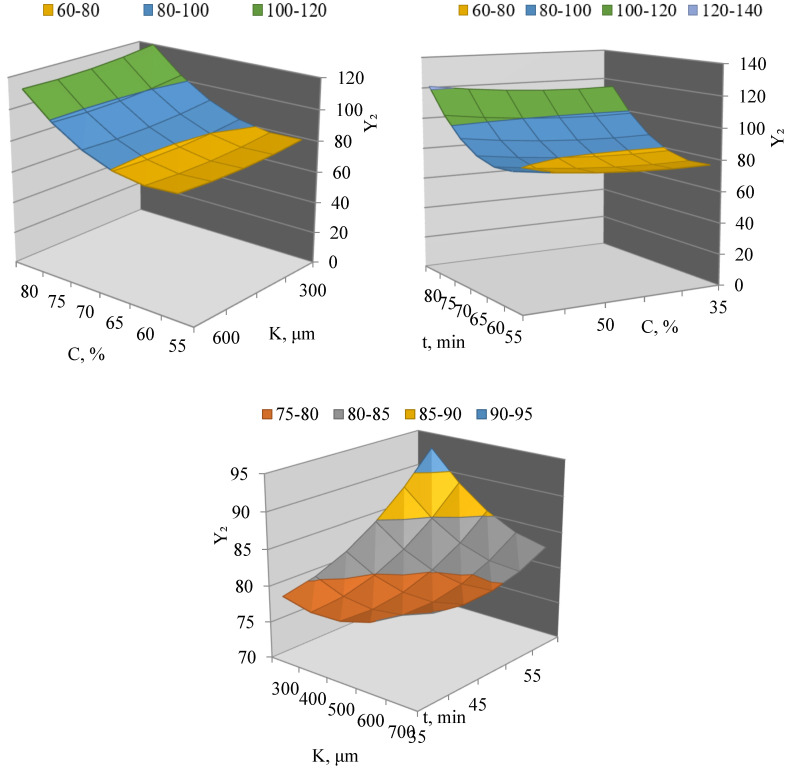
Three-dimensional response surfaces of total polyphenol content (Y_2_) depending on solvent concentration (C), particle size (K), and extraction time (t).

The multi-criteria optimization was performed using the desirability function approach, which combines the individual desirability values for extraction yield (maximization) and total polyphenol content (maximization) into an overall desirability index D. The maximum overall desirability achieved was D = 0.87 (on a scale from 0 to 1), confirming a high level of compromise between the two responses under the selected conditions (Figure 2).

Analysis of the three-dimensional response surfaces showed that the maximum polyphenol content in the extract is achieved at high solvent concentrations and moderate particle sizes and extraction times. The response surface in the “solvent concentration–particle size” plane demonstrates a pronounced increase in polyphenol content with increasing ethanol concentration and decreasing particle size, which is associated with improved solvent solubility and increased contact surface area.

The response surface in the “solvent concentration–extraction time” plane indicates the presence of an optimum, beyond which further increases in ultrasonic treatment time do not lead to a significant rise in polyphenol content. This may be due to reaching an equilibrium state in the extraction process or partial degradation of phenolic compounds during prolonged ultrasonic exposure.

Analysis of the combined effect of particle size and extraction time showed that maximum Y_2_ values are achieved at intermediate levels of both factors, while using excessively large particles or overly long extraction times leads to reduced efficiency of polyphenol extraction.

Based on the analysis of the mathematical model and response surfaces, the rational parameters of ultrasonic extraction that ensure maximum polyphenol content in the extract correspond to a solvent concentration of approximately 65–70%, particle size of about 400–500 μm, and extraction time of around 45–50 min. These results confirm that ultrasonic extraction is an effective method for obtaining polyphenol-rich extracts from peanut shells and allows targeted control over extract quality through optimization of technological parameters.

Using the developed regression models and response surface analysis, a multi-criteria optimization of the ultrasonic extraction process was performed using the desirability function approach to simultaneously maximize extraction yield and total polyphenol content. It was determined that the optimal process conditions correspond to an ethanol concentration of 69.75%, peanut shell particle size of 300 μm, and extraction time of 53 min. Under these conditions, the predicted extraction yield is 9.05% and the total polyphenol content is 95.15 mg GAE/g of dry extract.

After determining the optimal parameters for ultrasonic extraction of polyphenols from peanut shells, the obtained extract was used to develop a functional fermented dairy product. This approach was chosen due to the high biological activity of polyphenolic compounds and the need to evaluate their impact on the physicochemical and sensory properties of food systems.

To assess the effectiveness of the optimized extract in a dairy product, four yogurt variants were prepared, differing in the concentration of peanut shell polyphenol extract (PSE). The extract was added to the milk base at 0.25% (sample C1), 0.5% (sample C2), and 0.75% (sample C3). A control sample (C) was prepared without extract addition. The samples were incubated at 42–45 °C for 4–8 h and then stored at 4 ± 1 °C. The physicochemical properties of the yogurt were analyzed over 15 days of storage.

To evaluate the effect of peanut shell polyphenol extract on yogurt quality, changes in water-holding capacity, syneresis, pH, and titratable acidity during storage were evaluated. The results are presented in Table 1.

As seen from the data in Table 1 the water-holding capacity (WHC) of all yogurt samples decreased during storage, which is typical for fermented dairy products. However, the PSE-enriched samples demonstrated higher WHC values compared to the control sample at all storage stages. The highest WHC was observed in sample C2, indicating that a moderate concentration of the extract has an optimal effect on the formation of the protein–polyphenol matrix in the yogurt.

Syneresis increased in all samples during storage, but its intensity was significantly influenced by the concentration of PSE. The control sample exhibited the highest whey separation, whereas the addition of the extract reduced syneresis. The lowest values were recorded in samples C2 and C3, suggesting that polyphenols can stabilize the yogurt structure through interactions with milk proteins.

During storage, a gradual decrease in pH was observed in all samples, associated with the continued production of lactic acid due to the metabolic activity of starter microorganisms. The addition of PSE caused a slight reduction in initial pH values; however, the differences between the control and enriched samples remained moderate and within the typical range for yogurts. The most pronounced decrease in pH was noted in sample C3, which contained the highest extract concentration.

Titratable acidity increased in all samples over the 15-day storage period. The PSE-enriched samples exhibited higher acidity values compared to the control, which may be attributed both to the acidic nature of polyphenolic compounds and their potential influence on the activity of lactic acid bacteria. The highest titratable acidity was observed in sample C3, whereas sample C2 showed a balanced relationship between acidity and other physicochemical parameters.

The obtained results confirm that the peanut shell polyphenol extract, produced under the optimal conditions of ultrasonic extraction, can be successfully used for the development of functional yogurt. Moreover, a 0.5% extract concentration (sample C2) provides the best combination of physicochemical stability and potential functional value of the product.

## 3. Discussion

This study demonstrates that ultrasonic-assisted extraction (UAE) can be effectively optimized to recover polyphenolic compounds from peanut shells and that the resulting extract can be incorporated into yogurt to improve key physicochemical stability parameters during refrigerated storage. The discussion below integrates the obtained regression-based optimization results with evidence from published studies on polyphenol extraction technologies, peanut by-product valorization, and polyphenol–food matrix interactions.

The extract was characterized by total polyphenol content (Folin–Ciocalteu), but detailed profiling via HPLC-DAD or LC-MS was not performed in this study. Known major compounds in peanut shells include luteolin, phenolic acids, and proanthocyanidins (Imran A. et al., 2022; Tran T. N. et al., 2025) [2,9]. Future work should quantify individual phenolics (e.g., luteolin) and assess batch-to-batch reproducibility to link specific compounds to yogurt functionality.

The response surface methodology (RSM) models confirmed that ethanol concentration, particle size, and extraction time collectively govern both extraction yield (Y_1_) and total polyphenol content (Y_2_). The optimal conditions (69.75% ethanol, 300 μm, 53 min) maximized both responses simultaneously (9.05% yield; 95.15 mg GAE/g). Such outcomes align with the general conclusions summarized by Sridhar A. et al., who emphasized that intensified extraction methods (including UAE) can increase efficiency and produce higher-quality extracts, while the final performance depends strongly on solvent composition and particle size. In the present work, ethanol concentration emerged as the most influential factor, reflecting the need to match solvent polarity to phenolic compound solubility and to promote diffusion from lignocellulosic matrices.

While total polyphenol content was maximized under optimal conditions, the Folin–Ciocalteu method has known limitations, including interference and weak correlation with true antioxidant activity (Antony A. et al., 2022) [10]. Future experiments should include complementary antioxidant assays (e.g., DPPH, ABTS, FRAP) to better correlate extract quality with extraction parameters and yogurt bioactivity.

The strong role of particle size is consistent with mass-transfer theory: reducing particle size increases surface area and decreases diffusion path length, improving solute release. Similar observations were reported by Liao J. et al. [11], who optimized UAE of flavonoids from peanut shells and highlighted the importance of particle size and ethanol concentration as critical parameters. Likewise, Imran A. et al. [9] showed that ethanol-based UAE yields higher phenolic recovery and antioxidant activity than conventional extraction, supporting the selection of ethanol as a food-compatible solvent and the relevance of ultrasound intensification for peanut shell matrices.

The model behavior also indicates nonlinearity and factor interactions. In UAE systems, excessive time or harsh conditions can eventually reduce functional quality due to oxidative and radical-mediated degradation. This mechanistic explanation is consistent with the review by Dzah C. S. et al. [3], who noted that ultrasound power and extended processing may generate reactive species and, depending on system composition, can contribute to phenolic degradation beyond an optimum. Although the present study controlled temperature (<40 °C) to limit thermal effects, the observed curvature and optimal zone suggest that prolonged exposure may still reach a point of diminishing returns. The broader extraction literature also cautions that “more severe” conditions do not always translate into “better polyphenols,” since the measured total phenolic content can be affected by reaction products and assay limitations, as discussed by Antony A. et al. [10] in relation to thermally driven transformations and the Folin–Ciocalteu method.

From a sustainability and valorization perspective, the results support the concept that peanut shells are not merely waste but a functional raw material for bioactive recovery. This is in line with Dean L. L. [4], who described peanut by-products as significant sources of bioactive polyphenols with multiple applications, and with Toomer O. T. [5], who highlighted the scale of peanut processing residues and their potential use as antioxidant and functional ingredients. The present work extends these ideas by demonstrating a targeted, statistically optimized UAE route specifically aimed at food application.

The incorporation of the optimized peanut shell polyphenol extract (PSE/PSPE) into yogurt (0.25–0.75%) produced measurable improvements in physicochemical stability: water-holding capacity (WHC) increased and syneresis decreased compared with the control throughout 15 days of storage. These changes are technologically important because WHC and syneresis are among the most sensitive indicators of gel network integrity in fermented dairy systems.

The observed increase in WHC and decrease in syneresis are consistent with the known ability of polyphenols to interact with milk proteins. Polyphenolic compounds can form noncovalent interactions (hydrogen bonding, hydrophobic interactions) with caseins and whey proteins, strengthening the gel network, reducing serum separation, and enhancing moisture retention. Similar trends have been reported in other plant-extract-fortified yogurts: studies cited in the literature show that polyphenol-rich plant extracts can modify viscosity, WHC, and syneresis, with the magnitude depending on dose and phenolic profile. In this context, the present findings are aligned with the general direction of results discussed across polyphenol fortification research, including the broader observations summarized by Sridhar A. et al. regarding the importance of preserving extract quality for functional food integration.

In the storage study, pH decreased and titratable acidity increased across all samples, which is typical for yogurt due to continued post-acidification driven by starter cultures. The PSE-enriched samples showed acidity values that remained within typical yogurt ranges, indicating that polyphenol addition did not disrupt fermentation dynamics beyond expected behavior. This is important for process feasibility, since excessive acidification or inhibition of starter cultures would limit industrial applicability. The gradual changes in pH and acidity observed here support the suitability of the extract as a functional ingredient under refrigerated conditions.

Among the enriched samples, the 0.5% formulation (C2) showed the most favorable balance of WHC improvement and controlled syneresis reduction during storage, while higher extract loading (0.75%, C3) further reduced syneresis but was also associated with stronger acidity development and the lowest pH at day 15. This suggests a dose-dependent technological trade-off: increasing polyphenol concentration may further reinforce gel stability, but it may also intensify acidity changes due to the intrinsic acidity of phenolic compounds and/or their influence on microbial metabolism.

This pattern is consistent with the broader concept that an optimal fortification level exists where the functional benefit is maximized without compromising product stability or creating overly strong changes in fermentation-related parameters. In other food fortification contexts, dose-dependence is also reported: for example, Makangali K. et al. [12] showed concentration-dependent increases in antioxidant indicators when enriching honey with plant materials, while the strongest effects were achieved at higher levels, accompanied by changes in technological properties. Similarly, in extraction and functional applications, Sorita G. D. et al. [7] emphasized that the activity and functionality of polyphenol fractions depend on both concentration and processing strategy, and that stabilizing approaches (e.g., encapsulation) can be needed if higher doses compromise stability.

Technological feasibility and scalability: UAE under moderate temperature control and food-grade ethanol concentrations can be statistically optimized to produce polyphenol-rich extracts from peanut shells, supporting a practical route for by-product valorization.

Functional dairy application: The optimized extract improves yogurt structural stability during storage, as evidenced by WHC and syneresis behavior, indicating a real technological benefit for fermented dairy systems.

The observed improvements in WHC and reduced syneresis likely result from non-covalent interactions (hydrogen bonding, hydrophobic) between polyphenols and milk proteins (e.g., casein micelles), strengthening the gel network. However, this study did not include rheological measurements (G′, G″), microstructural analysis (confocal laser scanning microscopy or SEM), or protein aggregation studies. These techniques would provide direct evidence of polyphenol–protein complexation and network changes, as demonstrated in other polyphenol-fortified yogurts (Wróblewska B. et al., 2023). [13]. Such analyses are recommended for future research to elucidate mechanisms.

Waste-to-value approach: Using peanut shell extracts in food aligns with sustainability trends and the broader movement toward utilizing agro-industrial residues, an approach demonstrated in other matrices and applications (e.g., plant wastes for bioactive recovery and functional uses), as reported by Saad A. M. et al. and Jesus M. S. et al. [14,15], although in different end-use contexts. Overall, the findings confirm that peanut shells represent a viable source of functional polyphenols and that an RSM-guided UAE strategy can produce extracts suitable for incorporation into functional yogurt with improved physicochemical storage stability.

The total polyphenol content in the enriched yogurt samples was not quantified during storage in the present study, as the primary focus was on evaluating the technological impact of the extract on yogurt stability and structure (WHC, syneresis, pH, titratable acidity). Future research could include HPLC or spectrophotometric monitoring of polyphenol retention and potential interactions/binding with milk proteins over time, which may influence bioavailability and long-term antioxidant capacity.

## 4. Materials and Methods

### 4.1. Raw Materials and Reagents

Peanut shells (*Arachis hypogaea* L.) were obtained as a by-product of peanut processing from peanuts purchased at a local market in Astana, Kazakhstan. The shells were manually separated from the kernels and carefully cleaned of any mechanical impurities. Whole cow’s milk, used for subsequent yogurt preparation, was procured from a local supplier. Analytical-grade ethanol was used for the extraction process.

The peanut shells were washed with distilled water to remove surface contaminants and dried in a vacuum oven at 55 °C for 36 h until a constant weight was achieved. The dried material was ground using a ball mill and fractionated through sieves with different mesh sizes. The particle size of the powder was expressed in micrometers (μm) and selected according to the experimental design matrix. The resulting powder samples were stored in airtight containers in a desiccator at 5 °C until extraction.

### 4.2. Ultrasonic Extraction of Polyphenols

Polyphenolic compounds were extracted from peanut shells using the ultrasonic-assisted extraction (UAE) method. A measured amount of peanut shell powder was mixed with an aqueous–ethanol solvent of the specified concentration according to the experimental design. The solid-to-liquid ratio was kept constant in all experiments to eliminate its influence on the results.

Ultrasonic treatment was performed using an ultrasonic disperser at a constant power of 200 W and a frequency of 10 kHz. The extraction time was varied according to the experimental design. The process temperature was monitored and maintained below 40 °C to prevent thermal degradation of polyphenolic compounds. Upon completion of the ultrasonic treatment, the extracts were centrifuged to separate the solid residues, and the supernatant was subsequently filtered.

The obtained filtrates were concentrated using a rotary evaporator at 55 °C to remove the solvent. The concentrated extracts were then freeze-dried to obtain dry polyphenol-rich extracts. The dried extracts were weighed and stored in airtight containers at −20 °C until further analysis.

### 4.3. Preparation of Functional Yogurt

Whole cow’s milk was pasteurized at 85 °C for 30 min, cooled to 42–45 °C, and inoculated with a commercial yogurt starter culture containing Lactobacillus delbrueckii subsp. bulgaricus and Streptococcus thermophilus (typical ratio 1:1, inoculation level ~2–3% *v*/*v* or according to manufacturer’s instructions). Peanut shell polyphenol extract (PSPE) was added to the inoculated milk at concentrations of 0.25% (C1), 0.5% (C2), and 0.75% (C3) *w*/*w* prior to pouring into pre-sterilized glass cups (100–150 mL). A control sample (C) was prepared without extract addition.

The mixtures were gently stirred to ensure uniform distribution of the extract, sealed, and incubated in a thermostated incubator at 42–45 °C for 5–7 h until pH reached 4.6 ± 0.1 (end of fermentation). After coagulation, yogurts were rapidly cooled to 4 ± 1 °C and stored refrigerated for up to 15 days. Physicochemical properties were assessed at days 0, 5, 10, and 15.

### 4.4. Mathematical Model of the Influence of Technological Parameters on the Extraction Process

To identify and quantitatively assess the effect of technological parameters on the ultrasonic extraction of polyphenolic compounds from peanut shells, a mathematical model describing the process was developed. Regression analysis was employed to construct a second-order polynomial model. For experimental design, a second-order rotatable Box–Behnken design was applied with K = 3 factors.

The experimental plan included 15 runs, including three replicates at the central point, allowing for the assessment of reproducibility and experimental error. The total number of regression equation coefficients was 10. This design was chosen for its efficiency in optimizing technological processes and its ability to reveal both linear and quadratic effects of factors, as well as their interactions.

Based on preliminary experimental studies, the following independent variables were selected as having the greatest impact on extraction efficiency: solvent concentration (C, %), particle size of ground peanut shells (K, μm), and extraction time (t, min). Extraction yield (Y_1_, %) was used as an optimization criterion to characterize the technological efficiency of the process, while total polyphenol content (Y_2_, mg GAE/g) was used to reflect the quality of the obtained extract.

During the experiments, confidence intervals for all main parameters were determined, and mean values and standard deviations were calculated. The obtained experimental data were used to construct regression models and evaluate the significance of the factors.

Table 2 presents the natural and coded levels of factor variation, as well as the ranges used in the experiment.

The obtained experimental data were approximated using a second-order polynomial equation describing the dependence of the responses on the investigated factors. The adequacy of the model was evaluated using analysis of variance (ANOVA), the coefficient of determination (R^2^), and statistical significance testing of the regression coefficients. Optimal extraction process parameters were determined based on the maximization of the response variables (Table 3).

The experimental matrix was developed based on a second-order rotatable central composite design (CCD) for three factors (K = 3) with a star-point distance of α = 1.68. The design included eight factorial points, six axial points, and one central point, allowing for the evaluation of linear and quadratic effects, factor interactions, and the assessment of experimental reproducibility. The natural values of the factors were calculated based on the specified ranges and used for conducting experimental studies on the ultrasonic extraction of polyphenols from peanut shells.

Extraction yield (Y_1_) was calculated as the percentage ratio of the mass of the dry extract obtained after freeze-drying to the mass of the dry peanut shell sample used for extraction. The results were expressed as a percentage of dry matter.

Total polyphenol content (Y_2_) was determined spectrophotometrically using the Folin–Ciocalteu reagent. A weighed portion of the extract was dissolved in the appropriate solvent, and an aliquot of the solution was mixed with the Folin–Ciocalteu reagent and sodium carbonate solution. After incubation in the dark at room temperature, the optical density of the solution was measured using a UV–Vis. A calibration curve was constructed using gallic acid, and the results were expressed as milligrams of gallic acid equivalents per gram of dry extract (mg GAE/g).

### 4.5. Physicochemical Analysis of Functional Yogurt

#### 4.5.1. Determination of pH and Titratable Acidity

The pH of functional yogurt enriched with peanut shell polyphenol extract was measured using a digital pH meter (HI-2211, Hanna Instruments, Romania). For the analysis, 5 mL of yogurt sample was first diluted with 10 mL of distilled water and thoroughly mixed. The instrument was calibrated using three standard buffer solutions of different pH values.

Titratable acidity was determined by acid–base titration. A 10 g portion of yogurt was mixed with 10 mL of hot distilled water and titrated with 0.1 N sodium hydroxide solution in the presence of 0.5% phenolphthalein as an indicator until a stable pink color appeared.

#### 4.5.2. Water-Holding Capacity of Yogurt

The water-holding capacity (WHC) of yogurt was determined according to a method described in the literature (Sridhar A. et al. [1]) with minor modifications. Yogurt samples (20 g) were centrifuged at 5000 rpm for 10 min at 20 °C. After centrifugation, the separated whey was carefully removed, and the remaining gel was weighed. The water-holding capacity (WHC) was calculated using the following formula:

$$WHC\left(\%\right)=\frac{M-Mc}{M}\times 100$$

where **Mc** is the mass of whey separated after centrifugation, g; **M** is the initial mass of the yogurt sample, g.

For the determination of syneresis, yogurt samples were first held at 5 °C. Then, 25 mL of the formed gel was carefully transferred into 50 mL centrifuge tubes, minimizing disruption of the product structure. Centrifugation was carried out at 3394 rpm for 20 min using a CM-6MT centrifuge. Syneresis was expressed as the volume of supernatant per 100 g of yogurt (mL/100 g) and was calculated using the following formula:

$$Syneresis\;\left(\%\right)=\frac{Vc}{V}\times 100$$

where **Vc** is the volume of separated whey, mL; **V** is the initial volume of the yogurt sample, mL.

All experiments were performed in triplicate, and the results were expressed as mean ± standard deviation. Statistical analysis was carried out using response surface methodology (RSM). Analysis of variance (ANOVA) was applied to assess the significance of linear, quadratic, and interaction effects of the factors, as well as the adequacy of the obtained models. Optimal extraction conditions were determined based on the simultaneous maximization of extraction yield and total polyphenol content.

## 5. Conclusions

In this study, a comprehensive optimization of the ultrasonic extraction of polyphenolic compounds from peanut shells was carried out, followed by an assessment of their potential application in functional yogurt. The main extraction factors considered were solvent concentration, particle size of the raw material, and extraction duration. Response surface methodology was applied to model the process, enabling a quantitative description of the influence of technological parameters on extract yield and total polyphenol content.

Based on the developed second-order regression models and analysis of response surfaces, the optimal conditions for ultrasonic extraction were determined: ethanol concentration of 69.75%, peanut shell particle size of 300 μm, and extraction time of 53 min. Under these conditions, the predicted extract yield was 9.05% and the total polyphenol content reached 95.15 mg GAE/g of dry extract, demonstrating the high efficiency of the developed extraction process.

The use of the extract obtained under optimal conditions for yogurt enrichment demonstrated a positive impact on its physicochemical properties during storage. The fortified yogurt samples exhibited an increased water-holding capacity and reduced syneresis compared to the control, indicating improved structural stability of the product. Meanwhile, pH and titratable acidity values remained within the typical range for fermented dairy products, confirming the technological suitability of the extract for incorporation into yogurt systems.

In conclusion, the optimized UAE process (69.75% ethanol, 300 μm particle size, 53 min) effectively recovered a polyphenol-rich extract (9.05% yield, 95.15 mg GAE/g) from peanut shells, directly addressing the research gap in multi-criteria optimization for this specific by-product. Fortification of yogurt with this extract at 0.5% provided the most favorable improvements in structural stability—increased water-holding capacity and significantly reduced syneresis during 15-day storage—while maintaining pH and titratable acidity within acceptable ranges for fermented dairy products. These findings confirm that peanut shell polyphenol extract serves as a viable natural stabilizer and functional ingredient, supporting sustainable valorization of agro-industrial waste and contributing to the development of enhanced functional yogurts. Further research on sensory evaluation, polyphenol stability/bioavailability, and scale-up potential is warranted to facilitate industrial application.

Further studies are recommended to assess the retention and stability of polyphenolic compounds during prolonged storage, evaluate sensory properties and consumer acceptance of the fortified yogurt, investigate antioxidant and antimicrobial activity in the final product, as well as perform in vitro or in vivo bioavailability tests to fully confirm its functional potential.

## Figures and Tables

**Table 1:** Physicochemical characteristics of yogurt samples during storage.

Indicators	Samples		Storage Duration (Days)		
		0	5	10	15
**Water-holding capacity (%)**	C	67.25	66.85	64.61	62.31
	C1	69.18	67.5	65.21	62.77
	C2	72.23	69.94	67.98	66.12
	C3	70.87	68.98	67.9	65.74
**Syneresis** (%)	C	15.78	17.37	19.14	21.25
	C1	14.24	15.32	16.78	17.65
	C2	13.89	14.34	15.27	16.55
	C3	12.97	13.23	14.47	15.21
pH	C	4.69	4.66	4.62	4.55
	C1	4.67	4.63	4.54	4.49
	C2	4.65	4.62	4.56	4.49
	C3	4.61	4.57	4.49	4.37
**Titratable acidity**	C	0.75	0.82	0.85	0.89
	C1	0.81	0.86	0.95	1.02
	C2	0.91	0.98	1.04	1.13
	C3	1.05	1.12	1.17	1.25

**Table 2:** Coding of the intervals and levels of variation in the input factors.

Factor		Level of Variation					Range of Variation
Natural	Encoded	−1.68	−1	0	+1	+1.68	
*C*, %	*x*_1_	55	60	65	70	75	5
*K*, μm	*x*_2_	300	400	500	600	700	100
*t*, min	*x*_3_	35	40	45	50	55	5

**Table 3:** Experimental matrix of the second-order rotatable design for optimization of the extraction process.

Experiment Number	x_1_	x_2_	x_3_	C, %	K, μm	t, min
1	−1	−1	−1	60	400	40
2	−1	−1	+1	60	400	50
3	−1	+1	−1	60	600	40
4	−1	+1	+1	60	600	50
5	+1	−1	−1	70	400	40
6	+1	−1	+1	70	400	50
7	+1	+1	−1	70	600	40
8	+1	+1	+1	70	600	50
9	−1.68	0	0	55	500	45
10	+1.68	0	0	75	500	45
11	0	−1.68	0	65	300	45
12	0	+1.68	0	65	700	45
13	0	0	−1.68	65	500	35
14	0	0	+1.68	65	500	55
15	0	0	0	65	500	45

## Data Availability

The raw data that supported the findings of this study are available from the corresponding author, T.M.Ch., upon fair and reasonable request.
